# Increased breathlessness in post-COVID syndrome despite normal breathing patterns in a rebreathing challenge

**DOI:** 10.1038/s41598-025-11728-x

**Published:** 2025-07-29

**Authors:** Dina von Werder, Maria Aubele, Franziska Regnath, Elisabeth Tebbe, Dejan Mladenov, Victoria von Rheinbaben, Elisabeth Hahn, Daniel Schäfer, Katharina Biersack, Kristina Adorjan, Hans C. Stubbe, Katleen Bogaerts, Rudolf A. Jörres, Dennis Nowak, Omer Van den Bergh, Stefan Glasauer, Nadine Lehnen

**Affiliations:** 1 Institute of Medical Technology, Brandenburg University of Technology Cottbus- Senftenberg, Cottbus-Senftenberg, Germany; 2 Graduate School of Systemic Neurosciences, Ludwig-Maximilians-Universität München, Munich, Germany; 3 Department of Psychosomatic Medicine and Psychotherapy, TUM University Hospital, Technical University Munich, Munich, Germany; 4 TUM Graduate School, School of Medicine and Health, Technical University Munich, Munich, Germany; 5 University Hospital of Psychiatry and Psychotherapy, University of Bern, Bern, Switzerland; 6 Institute of Psychiatric Phenomics and Genomics (IPPG), LMU University Hospital, LMU Munich, Munich, Germany; 7 Department of Medicine II, LMU University Hospital, LMU Munich, Munich, Germany; 8 REVAL – Rehabilitation Research Center, Faculty of Rehabilitation Sciences, Hasselt University, Diepenbeek, Belgium; 9 Health Psychology, Psychology and Educational Sciences, University of Leuven, Leuven, Belgium; 10 Institute and Outpatient Clinic for Occupational, Social and Environmental Medicine, University Hospital, LMU, Munich, Germany; 11 Comprehensive Pneumology Center Munich (CPC-M), Member of the German Center for Lung Research (DZL), Munich, Germany; 12 Faculty of Health Sciences Brandenburg, Brandenburg University of Technology Cottbus- Senftenberg, Cottbus, Germany

**Keywords:** Fatigue, Respiratory signs and symptoms, Respiration, Perception, Cognitive neuroscience, Computational neuroscience, Sensorimotor processing

## Abstract

Severe symptoms in the absence of measurable body pathology are a frequent hallmark of post-COVID syndrome. From a Bayesian Brain perspective, such symptoms can be explained by incorrect internal models that the brain uses to interpret sensory signals. In this pre-registered study, we investigate whether induced breathlessness perception during a controlled CO_2_rebreathing challenge is reflected by altered respiratory measures (physiology and breathing patterns), and propose different computational mechanisms that could explain our findings in a Bayesian Brain framework. We analysed data from 40 patients with post-COVID syndrome and 40 healthy participants. Results from lung function, neurological and neurocognitive examination of all participants were within normal limits on the day of the experiment. Using a Bayesian repeated-measures ANOVA, we found that patients’ breathlessness was strongly increased (BF_10,*baseline*_=8.029, BF_10,*rebreathing*_=11636, BF_10,*recovery*_=43662) compared to controls. When excluding patients who hyperventilated (*N* = 8, 20%) during the experiment from the analysis, differences in breathlessness remained (BF_10,*baseline*_=1.283, BF_10,*rebreathing*_=126.812, BF_10,*recovery*_=751.282). For physiology and breathing patterns, all evidence pointed towards no difference between the two groups (0.307 > BF_10_ < 0.704). In summary, we found intact breathing patterns and physiology but increased symptom perception in patients with post-COVID syndrome.

**Supplementary Information:**

The online version contains supplementary material available at 10.1038/s41598-025-11728-x.

## Introduction

The post-COVID syndrome encompasses a wide range of debilitating symptoms that considerably impair quality of life for many patients. Symptoms can affect different organ systems with some of the most prevalent symptoms being fatigue, exercise intolerance, several types of pain and breathlessness^1–3^. Biomedical findings have provided insights into potentially underlying pathophysiological changes of the disease. However, these findings typically only emerge at the group level. In individual patients, the association between symptoms and measurable pathophysiological findings can vary considerably, and, in some cases, symptoms cannot be explained by standard clinical diagnostic findings^4–8^. This suggests that pathophysiological changes contribute to the manifestation of post-COVID syndrome but are not always sufficient to explain the symptoms.

The Bayesian brain theory offers a new explanatory perspective on how a divergence between symptoms and physiological body states could arise. Given that the body and its environment are constantly changing, the brain needs to continuously adapt behaviour and bodily processes^9^. To achieve this, the brain translates information provided by sensors about the current state of body and environment into adequate actions. These actions will change the body state and elicit new sensory input that is again detected by sensors and sent to the brain. Since the brain does not have direct access to body states, it needs to infer them based on the sensory data it receives. The processing and representation in the brain of signals originating in the body is termed interoception^10–12^. Next to direct control of bodily states, interoceptive signals also provide input to symptom perception^13^.

**Fig. 1:**
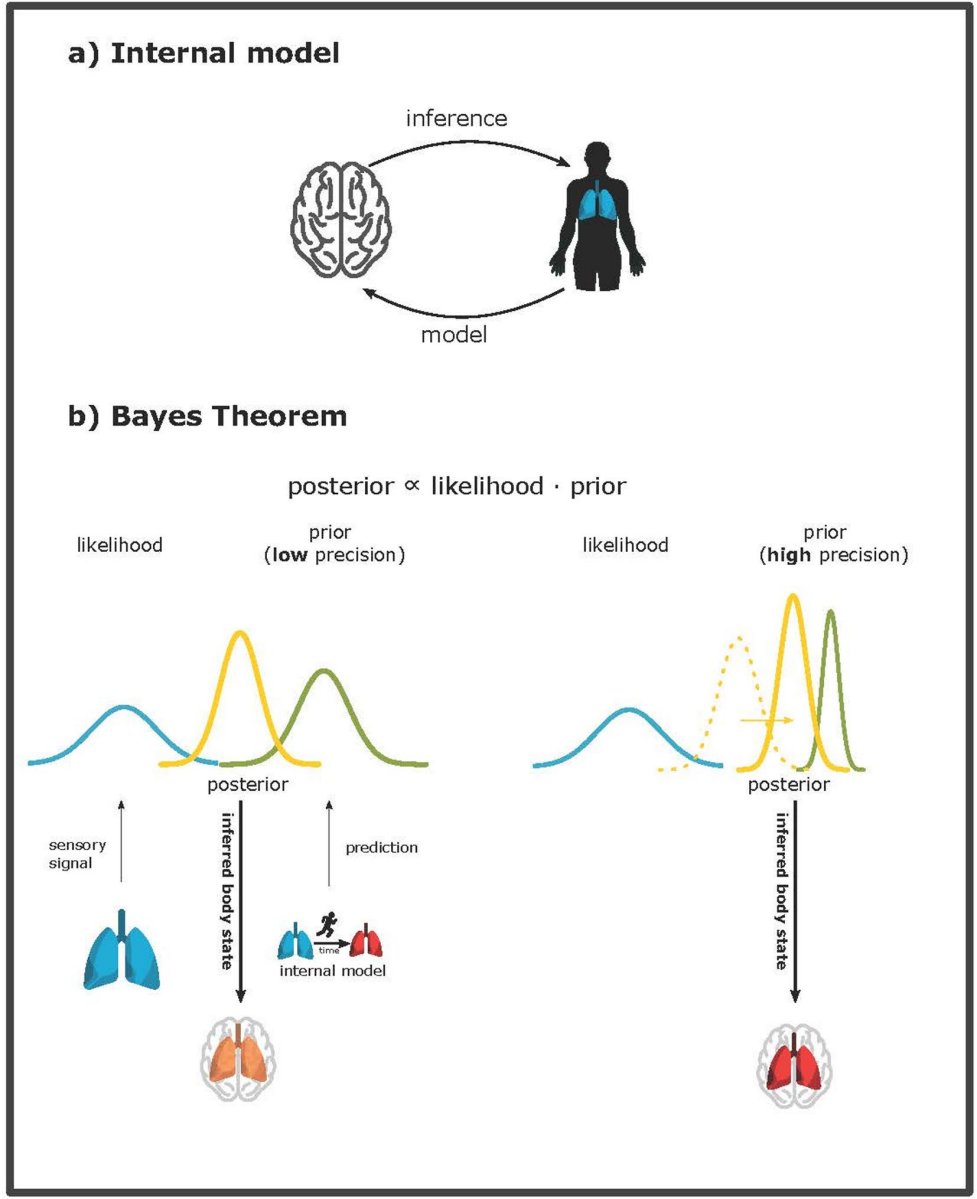
(**a)** Internal model and inference (based on Petzschner et al. (2017)). The brain holds a probabilistic model of how the received respiratory input has been generated. This includes the probabilities of different respiratory body states (prior) and the probabilities to observe the received respiratory input conditioned on the possible respiratory body states (likelihood). By inverting this model, the brain can infer the underlying respiratory state. This inference corresponds to applying Bayes theorem. (**b)** Bayes theorem describes how to optimally combine different sources of noisy data. It is commonly applied to interoception and symptom perception and suggests how the brain should combine noisy sensory (respiratory) data (likelihood) with model-based predictions (a priori knowledge, prior) to yield an optimal estimate of the underlying respiratory body state (posterior). Prior and likelihood are conceived as probability distributions to assign (un)certainty to each of these. The width (i.e., variance) of the likelihood function corresponds to the brain’s uncertainty (i.e., the signal’s reliability) only based on the sensory signal. The variance of the prior corresponds to the brain’s uncertainty of its knowledge about possible body states, before receiving any sensory signals. The variance of the resulting posterior corresponds to the brain’s uncertainty after combining the received sensory signal with prior knowledge. The posterior is shifted towards the distribution with less uncertainty (i.e., higher reliability). This means high certainty about the prior can shift the posterior more towards model-based predictions and away from sensory signals.

Symptom perception is often explained in a Bayesian brain framework. The Bayesian brain theory is based on Bayes’ theorem (see Fig. 1), which describes how to optimally combine noisy data (likelihood) with prior knowledge to estimate a hidden (body) state. Applied to brain function, it suggests that the brain uses implicit a-priori expectations (prior) to interpret sensory signals. These priors are based on general knowledge of body states and how they are influenced by context, which is represented in so-called internal models in the brain. Depending on the quality and associated reliability of both the sensory input (likelihood) and the prior, the resulting perception of symptoms (posterior) in consciousness can be closer to the sensory input or closer to the prior^14^. It has thus been suggested that highly reliable but incorrect priors (or very noisy sensory data) could explain persistent and strong symptoms even in the absence of any bodily dysfunction^15–17^, such as persistent breathlessness in the absence of underlying impairment of lung function^18–20^.

The Bayesian brain theory has also been applied to explain increased breathlessness ratings despite normal physiology and breathing patterns during a rebreathing challenge in functional breathlessness^21^, myalgic encephalomyelitis/chronic fatigue syndrome (ME/CFS) and fibromyalgia^22^. In these patient groups, breathlessness ratings during a rebreathing experiment were increased compared to healthy control participants, despite normal breathing patterns and physiology. Interestingly, this was only the case when participants (unknowingly) breathed room air and sensory input was low. When sensory input was strong during rebreathing, symptoms were similar to those of healthy participants and more strongly correlated with physiological measures^21–23^. These results point towards dysfunctions in the process of symptom generation due to incorrect internal models and thus inadequate priors, especially when the relevant sensory input is low.

In this study, we investigate whether similar differences in breathlessness perception during rebreathing are also present in patients with post-COVID syndrome with intact lung function and no signs of an underlying organic disease. We used the same rebreathing challenge as in these previous studies to perturb the respiratory body state in a controlled way and investigated how this influences the adaptation of the perceptual (breathlessness ratings) and physiological (heart rate and exhaled CO_2_) responses, as well as breathing patterns (respiratory rate and tidal volume). A strong association between symptom reports, breathing patterns and physiological measures is indicative of adaptive internal models that can correctly predict sensory signals. Conversely, a decoupling between those measures would indicate the strong influence of incorrect internal models leading to priors that bias symptom perception away from actual sensory input. In additional exploratory analyses, we investigated whether symptom perception during the experiment depends on whether patients are hyperventilating and whether patients experience breathlessness as part of their post-COVID syndrome or not. We hypothesize that, compared to healthy control participants, patients with post-COVID syndrome show:

I) Similar breathing patterns and physiology.
II) Increased breathlessness ratings before and after the rebreathing challenge.
III) Similar breathlessness ratings during rebreathing, i.e., during a period with a strong respiratory stimulus.
IV) Similar breathing patterns and reports of breathlessness in the subgroup of patients with and without a history of post-COVID breathlessness.

## Methods

The current study is part of the innovative training network ETUDE (Encompassing Training in fUnctional Disorders across Europe; https://etude-itn.eu/), ultimately aiming to improve the understanding of mechanisms, diagnosis, treatment and stigmatization of Functional Disorders^24^.

It was carried out in accordance with the Declaration of Helsinki and was approved by the ethics committee of the Technical University of Munich (2021 − 610_2-S-SR). All participants provided signed informed consent and received financial compensation of 10€ per hour.

We preregistered the study procedure and analysis on the Open Science Framework prior to data collection. It can be accessed under: https://osf.io/5ysn8. Data from five healthy control participants and five patients was previously used in a publication by our lab^20^.

### Participants

**Fig. 2:**
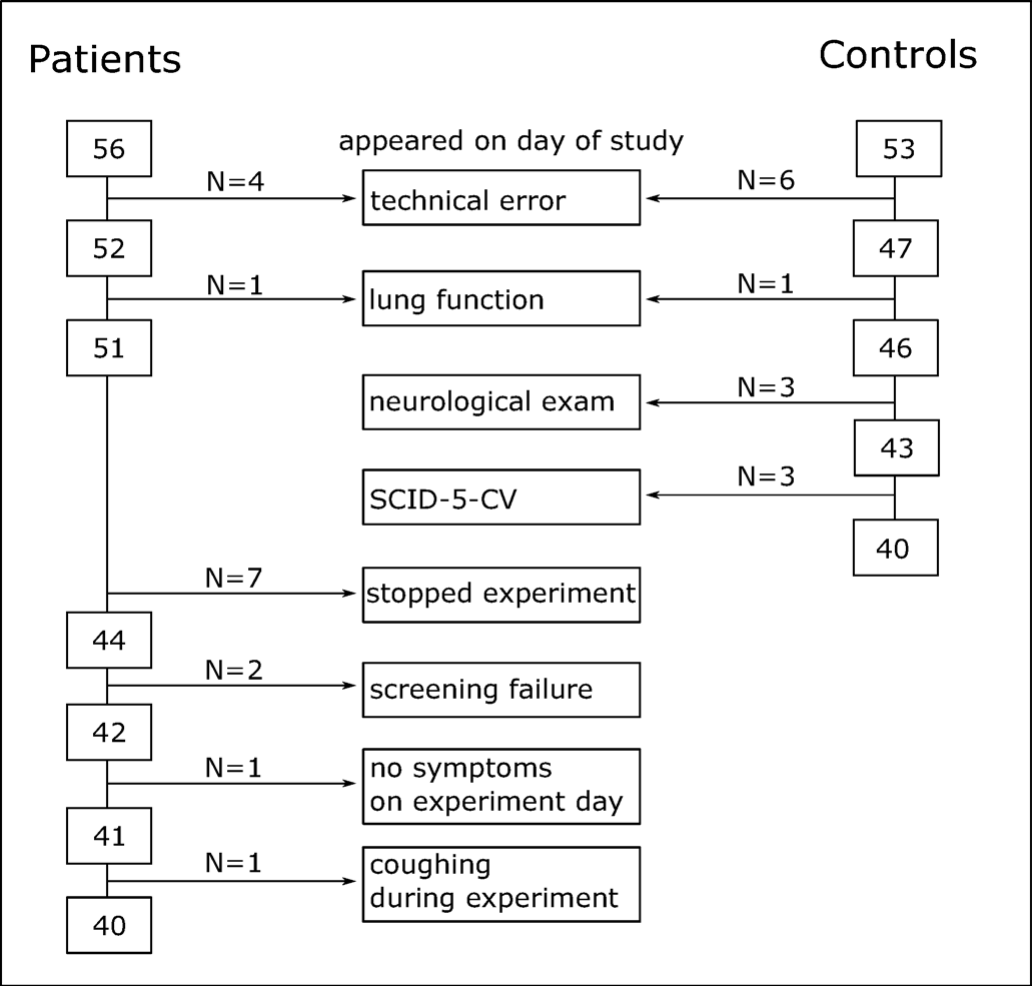
Participants that were excluded from data analysis.

Overall, 56 patients and 53 healthy participants were measured that met all inclusion and none of the exclusion criteria at the time of screening. Of these, 40 patients (M_age_ = 40.7 years, SD_age_ = 11.7 years, 23 women) and 40 healthy control participants (M_age_ = 37.35 years, SD_age_ = 12.55 years, 25 women) were included for final data analysis. See Fig. 2 for reasons of exclusion.

All individuals eligible for the current study were at least 18 years old, had sufficient German language skills to understand instructions and questionnaires and had a previous SARS-CoV-2 infection. Exclusion criteria for both groups were neurological, cardiological or pulmonological impairment, pregnancy and severe episode of major depression, severe psychosis or addiction disorder.

All patients had a post-COVID diagnosis from a specialized post-COVID centre at university hospital clinics and were severely affected by fatigue and/or breathlessness for at least 3 months. They were only included if according to the specialized post-COVID centres, no standard clinical tests revealed an explanation of their symptoms.

All healthy participants needed to be symptom free for at least 3 months after their last SARS-CoV-2 infection and were excluded if they were suffering from a functional (somatoform or dissociative) disorder.

### Study procedure

Measurements were conducted from July 2022 to May 2024. After written informed consent, all individuals performed the rebreathing experiment described below. Then participants filled out questionnaires and a detailed clinical characterization was performed. This was done after the rebreathing experiment as to not interfere with experimental results.

### Rebreathing experiment

The experiment was based on the standard rebreathing paradigm^25^ previously used to investigate breathlessness perception and breathing patterns in various patient groups and healthy participants^21,22^. Individuals wore a nose clip and breathed through a mouthpiece into a breathing circuit. The circuit was designed such that the experimenter could sit behind a visual barrier and let the participant either breathe normal room air or air from the rebreathing bag. Participants were blinded to the actual source of air breathed and its timing, and wore headsets so they were unable to hear when a valve switch was performed for the different breathing conditions.

To get accustomed to the breathing circuit and breathlessness rating scale, participants breathed into the circuit and were informed that they are breathing room air for the next 60s. During the last 30 s they additionally rated their breathlessness as described below. In the main session, participants were instructed that the CO_2_ concentration in inhaled air can change and that this might or might not lead to breathlessness. The exact wording (and an English translation) is available in the Appendix. The rebreathing experiment started with room air for 60 s, after which the valve was switched to the rebreathing condition. After 150 s the valve was switched back to room air for another 150s. Breathing flow, CO_2_ concentration in breathed air and heart rate were measured with a sampling rate of 50 Hz.

CO_2_ concentration in inhaled and exhaled air was measured with a sampling port at the mouthpiece that was connected to a side stream CO_2_ sensor (*Masimo*,* NomoLine*,* ISA CO*_2_). Respiratory flow was assessed with a heated pneumotachograph (0–400 L/min,*Hans Rudolph*). Heart rate and peripheral oxygen saturation were measured using an oximeter (*Nonin Xpod*) that was attached to the left index finger. Synchronized data recording from all devices and data storage was ensured by using the *SmartLab* Instrumentation system with *Insight Software* from *Hans Rudolph*. A three-way manual control valve (*Hans Rudolph*) allowed switching the source of breathed air between room air and rebreathing. The rebreathing bag was filled with a gas concentration of 5% CO_2_ and 95% O_2_ (*Carbogen*,* Linde*). Synchronization between breathlessness ratings and physiological data was evaluated by installing an *Arduino board* that detected mouse clicks and sent this information to the *SmartLab* Instrumentation system.

### Questionnaires

**Patient Health Questionnaire (PHQ-15)**: The somatic symptom scale of the Patient Health Questionnaire^26,27^ asks how bothered participants have been by 15 common somatic symptoms in the past two weeks. Ratings are on a three-point scale (0: not bothered at all, 1: bothered a little, 2: bothered a lot) and global ratings can range from 0 to 30.

**Chalder Fatigue Scale (CFQ)**: The Chalder Fatigue Scale^28,29^ comprises 11 items and measures the extent and severity of fatigue. Questions 1–7 measure physical fatigue and questions 8–11 cognitive fatigue. Ratings are on a four-point scale (0: better than usual, 1: no worse than usual, 2: worse than usual, 3: much worse than usual). The Likert scoring method was applied, so global scores can range from 0 to 33.

#### Common post-COVID symptoms:

All participants were presented with a list of seven symptoms commonly reported in post-COVID syndrome and were asked to rate which of them they are currently experiencing and the respective level of severity. Ratings are on a four-point scale (0-not present, 1-mild, 2-moderate, 3-severe).

#### Concurrently perceived breathlessness:

During the experiment participants’ breathlessness ratings and their timepoints were recorded with a program written in OpenSesame, version 3.3.11^30^. It presented a scale with every integer from 0 to 100 every 10s. A tone marked the start of a new rating. Next to the scale verbal descriptions of the breathlessness label (as in Bogaerts et al. (2010)) were given (0 – no breathlessness, 5 – barely noticeable, 10 – very slight, 20 – slight, 30 – moderate, 40 – rather strong, 50 – strong, 60–80 very strong, 90 – very, very strong, 100 – not bearable; translations from German).

### Clinical characterization

On the day of the experiment, we performed a detailed clinical characterization to ensure that participants met all inclusion and none of the exclusion criteria.

**Pulmonary function** was assessed in the Department of Occupational Medicine (Ludwig-Maximilians University) using spirometry and diffusion capacity (*Masterscreen PFT*,* Vyaire*) according to the European Respiratory Society (ERS) clinical guidelines. The Global Lung Function Initiative (GLI) calculator was used to evaluate the percentage of predicted norm value and percentage of LLN for each participant depending on age, height and sex. The reference equations in the GLI calculator for spirometry are based on^58^, for diffusion capacity on^59^ and for lung volumes on^60^. All equations were based on Caucasian data. We determined the following parameters: FEV1 – forced expiratory volume in 1 s. FVC – forced vital capacity. DLCO – diffusion capacity for carbon monoxide. KCO – carbon monoxide transfer coefficient. RV - residual volume. VA – alveolar volume.

The **semi-structured clinical interview for the diagnosis of DSM-5 disorders**^31^ (SCID-5-CV) was performed to ensure that none of the exclusion criteria were met.

**A standardized neurological examination** was performed to rule out neurological impairment.

The **Montreal Cognitive Assessment**^32^ (MOCA) was performed to rule out severe neurocognitive impairment. The MOCA assesses different cognitive domains including short-term memory, visuospatial abilities, working memory, attention, language, abstract reasoning, and orientation. Scores can range from 0 to 30. Scores of 26 and above indicate normal cognitive abilities, scores between 6 and 25 indicate at least mild cognitive impairment and scores below 6 severe cognitive impairment.

**Missing data and exclusion on the day of study**

Participants were excluded for data analysis if they showed signs of obstructive lung function on the day of the experiment, i.e., FEV1/FVC was below the lower limit normal^33^ (LLN), which is the 5th percentile of a healthy, non-smoking reference population. This was the case for one control participant and one patient. In another two control participants and three patients FEV1/FVC was within 2% lower than the LLN. In all these cases, a specialized pulmonologist visually inspected the lung function curves and decided that no obstructive lung function impairment was present, and these participants were included in the study. For further reasons of exclusion for data analysis after appearance on the day of study, see Fig. 2.

Respiratory flow, heart rate and CO_2_ concentration in breathed air, as well as breathlessness ratings are available for all included participants, except one patient. Due to technical errors, for this patient only CO_2_ recordings and breathlessness ratings, but not respiratory flow data are available.

The CFQ was added after study start to characterize fatigue levels in patients in more detail. At this time point, we had already measured 15 healthy control participants and one patient. The CFQ is thus missing for 15 healthy participants and one patient. However, since this questionnaire only serves to characterize fatigue levels in patients, we still show CFQ scores in the results section. Due to experimenter error, the MOCA is missing for one healthy control participant.

### Data processing

Data were processed using Python 3.0. Respiratory flow measurements were calibrated with calibration pumps and normalized to participants’ height by dividing by their height in meters. Breath-by-breath data were obtained by assessing timepoints of in- and exhalation as defined by the zero crossings of the respiratory flow data. For each breath, the durations of inspiration (T_i_) and expiration (T_e_) were determined, and the inspiratory and expiratory volumes (V_i_ and V_e_) were calculated by taking the integral of the respiratory flow signal. Respiratory rate (RR) and tidal volume (Vt) were calculated as follows:

$$\:RR=\frac{60}{{T}_{i}+{T}_{e}}$$

$$\:Vt=\frac{{V}_{i}+{V}_{e}}{2}$$

Fractional end-tidal CO_2_ (FetCO_2_) was approximated by taking the maximum concentration of the exhaled CO_2_ concentration in each breath. FetCO_2_ concentration serves as a proxy for arterial CO_2_ concentration^34^. We thus captured breathing patterns (RR and Vt) as well as the physiological response (heart rate, FetCO_2_) which serve as a proxy of the sensory stimulus for mechano- and chemoreceptors.

**Fig. 3:**
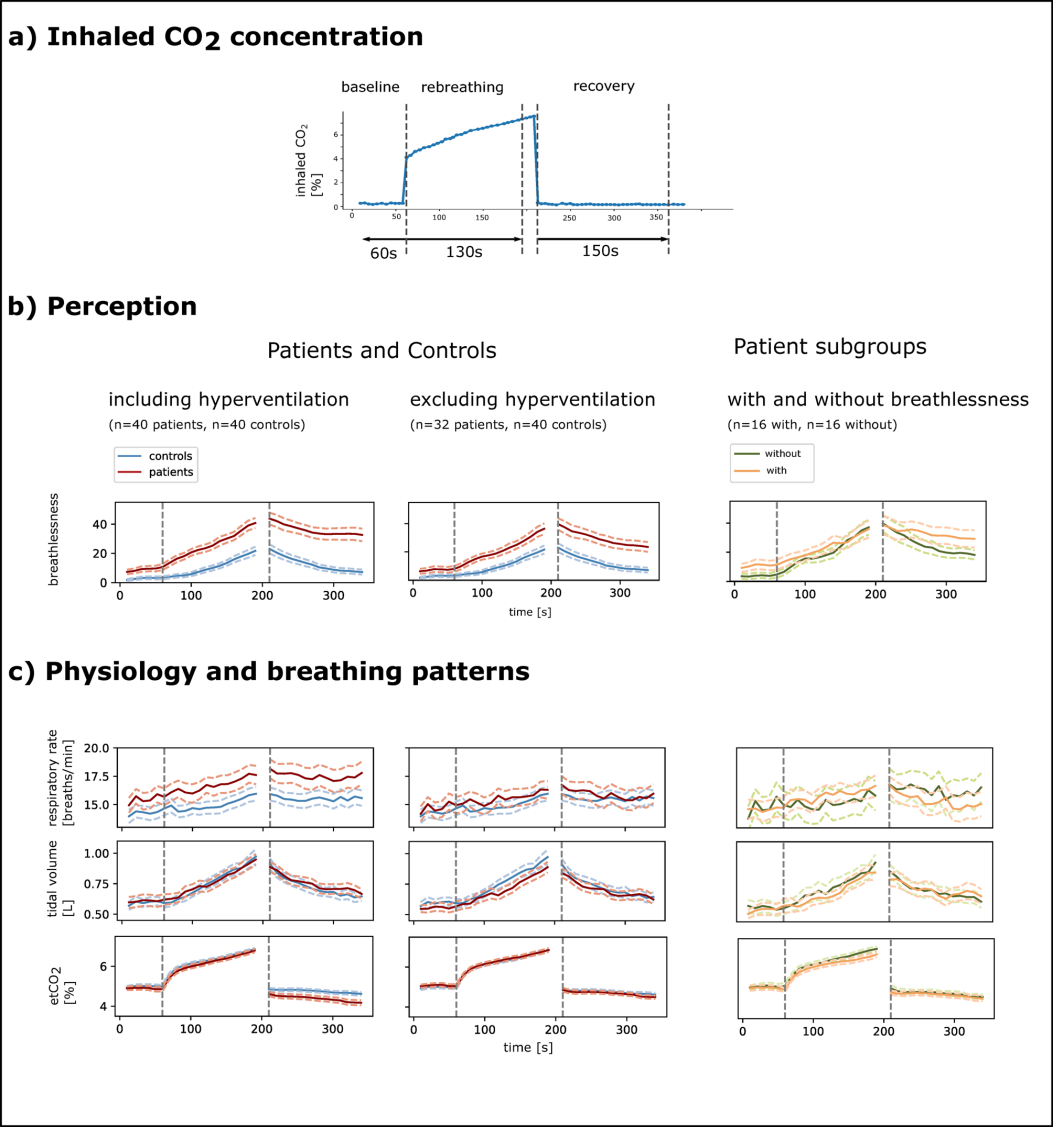
Results rebreathing experiment. (**a**) Exemplary time course of inhaled CO_2_ concentration from one individual. First, participants breathe room air, i.e., inhaled CO_2_ concentration is low. After a valve switch, they rebreathe from a rebreathing bag, initially filled with 5% CO_2_ and 95% O_2_. This constitutes a strong respiratory stimulus. After 150 s the breathing circuit is opened again, and participants breathe room air. The exact rebreathing and recovery start was defined based on the inhaled CO_2_ concentration of each individual (see Methods). (**b**) Differences in breathlessness reports for all patients and healthy participants (left), only those that were not hyperventilating (middle) and patients with versus without breathlessness that were not hyperventilating (right). (**c**) Breathing patterns and FetCO_2_ for all three group comparisons. Solid lines – mean, dashed lines – standard error of the mean.

Breathlessness ratings were interpolated to match the breath-by-breath data of each participant. Each breath was defined to start with an inhalation that is followed by an exhalation. Breath-by-breath data were then averaged over 10 s intervals. The start of the rebreathing phase was defined as the time point of the start of the breath that followed the valve switch. The start of the recovery phase was defined as the time point of the start for the breath that followed the valve switch back to room air. The timepoint that participants first and last inhaled an increased CO_2_ concentration during rebreathing depends on the individual’s respiratory rate and timepoint of the valve switch in the breathing cycle. Thus, although the valve switch occurred at 60 s and 210 s, the duration of inhaling increased CO_2_ concentration during rebreathing was slightly lower for some participants. We thus analysed the first 130 s after the rebreathing start to ensure that data is available for all participants. The baseline phase was defined as the 60 s before the rebreathing start and the recovery phase as the 150 s after recovery start (see Fig. 3).

### Statistics and reproducibility

Statistical tests were carried out in JASP^35^. To compare demographic characteristics and questionnaire scores, a Bayesian independent samples t-test (two-sided) was used. To evaluate possible group differences in the rebreathing experiment, we performed a Bayesian repeated-measures ANOVA for each of the different conditions (baseline, rebreathing, recovery) with respectively RR, Vt, FetCO2, heart rate and breathlessness rating as the dependent variable, the between factor group (main analysis: patients versus healthy participants; exploratory analysis: post-COVID with breathlessness versus post-COVID without breathlessness) and the independent within-factor time segment (repeated measures factor). We used a uniform prior over all models. We adopted a Bayesian statistical approach that allows to quantitatively make statements about whether the null or alternative hypothesis are more likely^36^. The Bayes factor 10 (BF_10_) quantifies the ratio between the alternative hypothesis given the data and the null hypothesis given the data. For example, a BF_10_ = 5 indicates that the alternative hypothesis is 5 times more likely than the null hypothesis, whereas a BF_10_ = 1/5 indicates that the null hypothesis is 5 times more likely. BFs of 1,]1–3],]3–10],]10–30],]30–100] and > 100 are often interpreted as “no”, “anecdotal”, “moderate”, “strong”, “very strong”, and “extreme evidence” (Lee & Wagenmakers, 2014)^37^ or according to Jeffreys (1961)^38^ “not worth more than a bare mention”, “substantial”, “strong”, “very strong” and “decisive evidence”. In our interpretation of Bayes Factors, we follow Lee & Wagenmakers (2014)^37^.

The required sample size for our study was estimated based on the effect sizes of two previous studies using the same rebreathing paradigm^21,22^. Using G*Power for the a-priori power analysis, we obtained a required sample size of 34 participants per group. We included a safety margin and thus measured 40 participants. The detailed sample size calculation can be found in the preregistration (https://osf.io/5ysn8).

### Exploratory analyses

Since hyperventilation has previously been described as a frequent breathing pattern in patients suffering from post-COVID syndrome^39^, we investigated whether this is also the case in our experiment. Since there is currently no gold standard to diagnose dysfunctional breathing^40^ nor a specific cut-off defined for hyperventilation, we defined hyperventilation in our study as an FetCO_2_ level of < 3.5%, which is in the range of FetCO_2_ levels previously shown to cause symptoms in healthy individuals^41^. We subsequently compared the subset of patients that was not hyperventilating to the healthy control group.

We further investigated whether experiencing breathlessness as part of post-COVID syndrome influences the experienced breathlessness ratings during the experiment. For this purpose, we compared all patients that reported at least mild breathlessness in the COVID symptom questionnaire (see Table 2) to those who reported to not suffer from breathlessness.

For both exploratory analyses, we used the same Bayesian repeated-measures ANOVA as described above.

## Results

### Demographic and clinical characteristics

We report on a sample of 40 patients who suffer from post-COVID syndrome without clinical signs of cognitive, neurological or pulmonological impairment and 40 healthy control participants. Both groups had a similar distribution of age, sex, and body mass index (Table 1 and Appendix Figure A). The infection that led to post-COVID symptoms was between March 2020 and December 2022. On average patients have suffered for 18.02 months (range: 5–36 months) from symptoms at the time of study participation. Patients report very high levels of fatigue as measured with the CFQ and more bodily symptoms than healthy participants as measured with the PHQ-15 (see Table 1). According to the PHQ-15 results, patients are bothered most by ‘Feeling tired or having low energy’, ‘trouble sleeping’ and ‘headaches’ (see Appendix Figure B). When asked about current post-COVID symptoms, patients most often reported fatigue, followed by difficulties concentrating, tiredness and dizziness or light-headedness (see Table 2).

There is no supporting evidence indicating different lung function parameters between healthy control participants and patients, however, there was anecdotal evidence for an increase in DLCO and KCO (see Table 3). It has previously been shown, that the odds of experiencing any respiratory symptom decreases the higher the ratio of FEV1/FVC and reaches a minimum at 80%^42^. All our participants were above that threshold.

None of the included participants has a neurological impairment or severe neurocognitive impairment. Three patients and two healthy participants have MOCA scores below 26, suggesting mild cognitive impairment (see Table 3).

**Table 1:** Demographic and clinical characteristics. Chalder fatigue scale was available for 39 patients and 25 healthy control participants. Possible scores CFQ (0–33) and PHQ-15 (0–30). BMI – body mass index, CFQ – Chalder fatigue scale, PHQ-15 – Patient health questionnaire 15.

	Post-COVID		Control group		BF_10_
	Mean	Range	Mean	Range	
Age (years)	40.7	(22–62)	37.35	(24–65)	0.442
Sex (female/male)	23/17	n.a.	25/15	n.a.	n.a.
BMI (kg/m^2)	24.13	(18–34)	23.68	(19–35)	0.265
Symptom duration (months)	18.02	(5–36)	n.a.	n.a.	n.a.
CFQ	25.15	(11–33)	9.96	(2–18)	3.80*10^16^
PHQ-15	12.75	(4–24)	3.3	(0–10)	1.79*10^12^

**Table 2:** Prevalence and severity of symptoms commonly reported in post-COVID. Severity was rated on a scale from 0 (not present) to 3 (severe). Mean severity for those participants that reported that the symptom was present. Data is available for 40 patients and 39 healthy control participants.

Symptom	Post-COVID			Control group		Std
	Frequency	Mean severity (1–3)	Std	Frequency	Mean severity (1–3)	
Fatigue	97.5%	2.36	0.66	20.51%	1.25	0.66
Difficulties concentrating	90.0%	2.06	0.74	15.39%	1.00	0.00
Tiredness	87.5%	2.23	0.76	28.21%	1.45	0.66
Dizziness/light headedness	70.0%	1.39	0.62	5.13%	1.00	0.00
Pain	65.0%	1.62	0.74	10.26%	1.25	0.43
Breathlessness	55.0%	1.64	0.64	0.00%	-	-
Loss of taste and/or smell	22.5%	1.89	0.74	2.56%	1.00	0.00
Nausea and/or vomiting	15.0%	1.00	0.00	0.00%	-	-

**Table 3:** Lung function parameters and results from the Montreal cognitive assessment (MOCA). % predicted – percentage of predicted norm value based on age, height and sex. % LLN – percentage of lower limit normal. FEV1 – forced expiratory volume in 1 s. FVC – forced vital capacity. DLCO – diffusion capacity for carbon monoxide. KCO – carbon monoxide transfer coefficient. RV- residual volume. VA – alveolar volume.

	Post-COVID		Control group		BF_10_
	Mean	Range	Mean	Range	
MOCA	28.26	(23–30)	28.46	(24–30)	n.a.
**FEV1/FVC** [% predicted]	96.59	(85.50–116.29)	97.27	(84.33–112.73)	0.255
[% LLN]	112.04	(98.97–134.81)	112.77	(97.61–130.22)	0.251
**FEV1** [% predicted]	99.41	(67.56–129.00)	101.18	(73.28–124.71)	0.281
[% LLN]	126.31	(86.17–169.65)	128.16	(98.34–166.81)	0.261
**FVC** [% predicted]	102.53	(77.90–129.95)	103.59	(79.69–131.18)	0.249
[% LLN]	129.92	(99.26–163.75)	130.98	(99.7–174.77)	0.242
**DLCO** [% predicted]	96.33	(67.88–130.09)	102.18	(83.19–127.08)	1.477
[% LLN]	123.19	(87.56–167.65)	130.18	(105.15–160.18)	1.228
**KCO** [% predicted]	97.35	(75.33–127.01)	103.78	(74.10–125.00)	1.794
[% LLN]	124.18	(95.64–161.93)	132.20	(98.22–160.68)	1.623
**RV** [% predicted]	104.71	(70.45–210.18)	106.69	(65.66–179.00)	0.246
[% LLN]	194.12	(124.37–403.79)	203.81	(130.44–374.57)	0.307
**VA** [% predicted]	99.00	(80.89–125.10)	99.00	(80.69–123.24)	0.232
[% LLN]	120.67	(99.14–153.33)	120.46	(97.93–149.87)	0.233

### Rebreathing experiment

The results of the rebreathing experiment are visualized in Fig. 3; Table 4. For a comparison of the Bayesian to a frequentist statistical approach, see Appendix Table A.

### Baseline

In the baseline phase with room air, reported breathlessness is higher in patients (M_patient_ = 9.0) than in healthy participants (M_healthy_ = 2.8; BF_10_ = 8.029, moderate evidence). There is anecdotal evidence for similar breathing patterns, i.e., respiration rate (BF_10_ = 0.817) and tidal volume (BF_10_ = 0.617) and a similar physiological response, i.e., FetCO_2_ concentration (BF_10_ = 0.695) and heart rate (BF_10_ = 0.671) in both groups.

### Rebreathing

During rebreathing patients report higher breathlessness (M_patient_ = 26.5) than healthy participants (M_healthy_ = 11.1; BF_10_ = 11636, extreme evidence). There is anecdotal evidence for similar tidal volume (BF_10_ = 0.361) and FetCO_2_ concentration (BF_10_ = 0.437) and anecdotal evidence for increased respiratory rate (BF_10_ = 2.007) in patients.

### Recovery

During recovery with room air, patients report higher breathlessness (M_patient_ = 36.6) than healthy control participants (M_healthy_ = 13.5; BF_10_ = 43662, extreme evidence). There is anecdotal evidence for an increased respiratory rate (BF_10_ = 1.877) and moderate evidence for decreased FetCO_2_ concentration in patients (BF_10_ = 5.018). Evidence for similar tidal volume (BF_10_ = 0.423) is anecdotal.

**Table 4:** Bayes factors (BF_10_) for different group comparisons.

	Baseline	Rebreathing	Recovery
**Patients and control group** Including hyperventilation			
Breathlessness	8.029	11,636	43,662
Respiration rate	0.817	2.007	1.877
Tidal volume	0.617	0.361	0.423
FetCO_2_	0.695	0.437	5.018
Heart rate	0.671	0.708	0.776
Excluding hyperventilation			
Breathlessness	1.283	126.812	751.282
Respiration rate	0.562	0.651	0.669
Tidal volume	0.619	0.640	0.478
FetCO_2_	0.704	0.307	0.480
Heart rate	0.568	0.500	0.531
**Patients with versus without post-COVID breathlessness**			
Breathlessness	1.326	0.414	0.618
Respiration rate	0.701	0.605	0.665
Tidal volume	0.664	0.480	0.512
FetCO_2_	0.689	0.626	0.684
Heart rate	0.797	1.336	1.632

### Exploratory analyses

When excluding patients that are hyperventilating from the group comparison, all evidence points towards similar breathing patterns (BF_10_ < 0.704), while evidence for increased breathlessness during rebreathing and recovery phase remains strong (BF_10_ > 126.812) and is anecdotal in the baseline phase (BF_10_ = 1.283).

We further investigated whether experiencing breathlessness as a post-COVID symptom leads to higher induced breathlessness perception during the experiment. For almost all phases, evidence for breathing patterns and breathlessness perception points towards similarity between groups and there is anecdotal evidence (BF_10_ = 1.326) for increased breathlessness perception in patients with post-COVID breathlessness in the baseline phase with room air (see Fig. 3 on the right and Table 4).

## Discussion

### Summary

We used a rebreathing experiment to perturb the respiratory body state in a controlled way. Breathlessness ratings in patients with post-COVID syndrome were increased compared to healthy individuals, while breathing patterns and physiology were similar. During rebreathing and recovery phase, there was no supporting evidence for higher breathlessness ratings in patients that experience breathlessness as part of their post-COVID syndrome and those who do not. 20% of patients hyperventilated during the experiment, however, differences in breathlessness remained when removing these patients from analysis.

### Intact breathing patterns

Our results are in line with our first hypothesis that stated similar adaptation of respiratory patterns and physiology to the rebreathing challenge in patients and healthy participants. This suggests that patients in our study were able to correctly process sensory signals related to respiration, infer the underlying respiratory body state and plan adaptive breathing patterns. As in several previous studies^4,5,8^, we find intact lung function in patients with post-COVID syndrome. Our results of similar breathing patterns, extend the common finding of intact lung function, by showing that not only mechanical ventilation and gas exchange in the lung are functioning appropriately, but also the sensory-control loop for breathing regulation is intact. It must be mentioned that while in all breathing conditions evidence pointed towards similar breathing patterns and physiology, evidence remained anecdotal. In addition, while most evidence pointed towards similar lung function measures between healthy participants and patients, we also observed anecdotal evidence for decreased lung diffusing capacity (DLCO and KCO) in patients. Our sample size might thus have been too small to detect subtle differences in breathing patterns or lung function. However, it remains questionable whether such small differences could explain the observed large differences in breathlessness ratings in our study.

### Dysfunctions in symptom perception

We were further able to confirm our second hypothesis that breathlessness is increased before and after the rebreathing challenge, i.e., during phases with room air. However, we also found strongly increased breathlessness during the rebreathing challenge, i.e., when the respiratory stimulus was very strong. This is against our third hypothesis and not in line with previous results in ME/CFS, fibromyalgia^22^ and functional breathlessness^21^.

One possible explanation for the latter divergent result is that the effect of rebreathing on breathlessness ratings is dependent on stimulus strength. While we used the same experimental paradigm as in other studies, FetCO_2_ levels in our study were lower (mean FetCO_2_ at end of rebreathing 6.9%) than in these studies (mean FetCO_2_ at end of rebreathing ~ 8% in^21^). This could either be due to differences in the technical setup (e.g., different dead space of the breathing circuit) or differences in breathing patterns. Slower and shallower breathing, as already exhibited during the baseline phase in participants in our study, leads to less CO_2_ that is exhaled into the breathing circuit during rebreathing and thus a lower respiratory stimulus intensity. This interpretation is consistent with the results of another study with non-clinical high and low symptom reporters using the same rebreathing paradigm^43^. Here, high symptom reporters also reported higher breathlessness during the rebreathing phase than low symptom reporters. Interestingly, in this study, FetCO_2_ levels were in the same range (mean FetCO_2_ at end of rebreathing ~ 7.3%) as ours.

### Potentially transdiagnostic mechanism

Furthermore, we could partly confirm our fourth hypothesis, i.e., breathlessness ratings are similar in post-COVID patients with and without a history of breathlessness. While breathlessness ratings during baseline were slightly increased in patients with post-COVID breathlessness, our statistical analysis yielded no group differences during rebreathing and recovery. This is in line with previous studies showing increased symptom reporting in different groups of patients regardless of their primary symptoms and suggests a more general dysfunction of symptom processing underlying persistent symptoms across diseases. Context and learned associations play a dominant role in symptom perception. Experimental setups such as the rebreathing experiment in this study and instructions that focus on symptom ratings create a context that activates symptom priors. This might be especially strong in patients and could outweigh symptom- or disease-specific processes, leading to the observed similarities across different studies, diagnoses and symptoms.

### Possible computational mechanisms

**Fig. 4:**
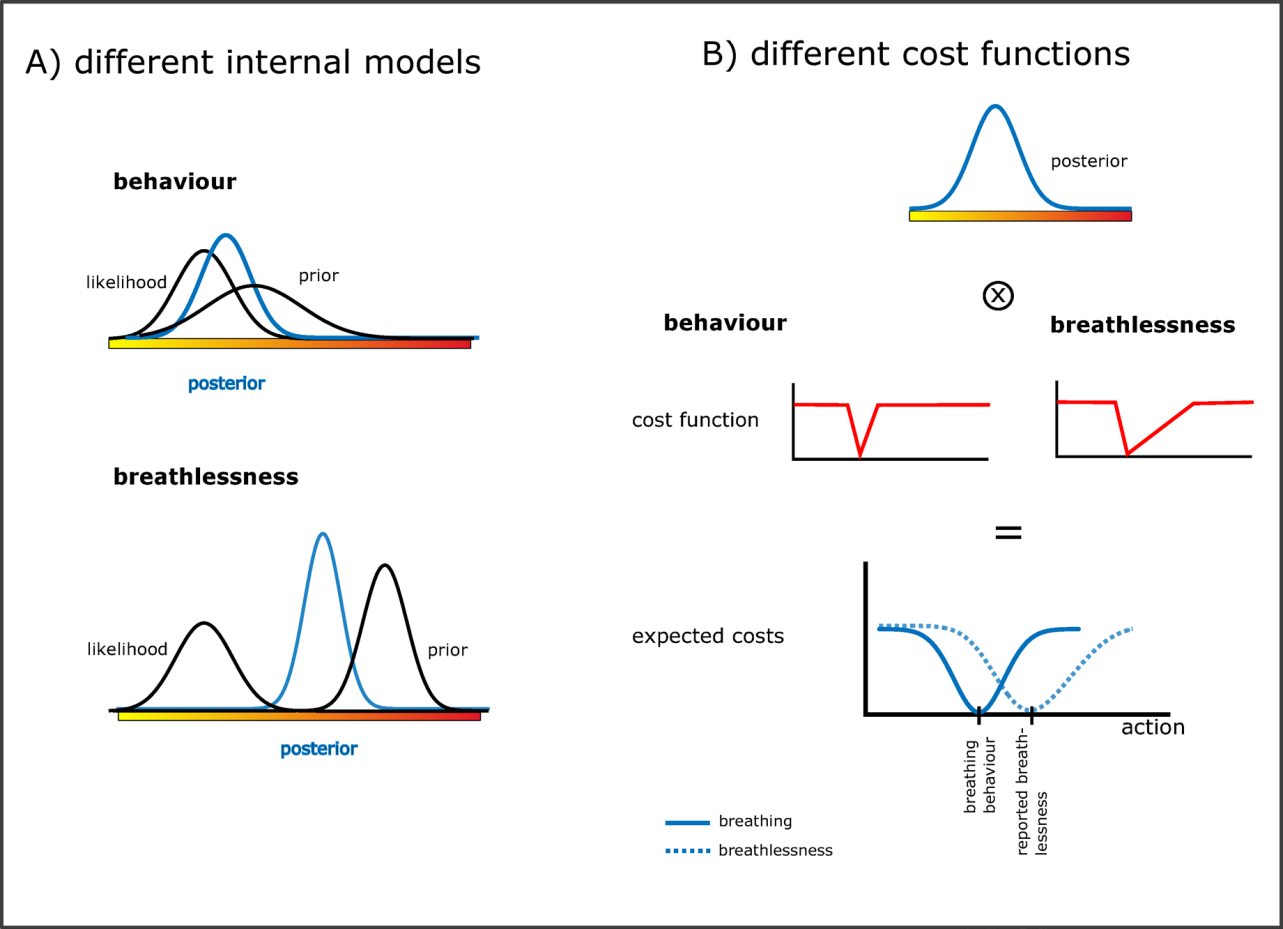
Possible computational mechanisms that could explain increased breathlessness despite normal breathing patterns. (**A**) Different internal models for breathlessness perception and breathing patterns. The same sensory input and its reliability is assumed for breathing patterns and breathlessness perception, represented as the same likelihood function. However, for breathlessness a higher and more reliable prior (i.e., higher mean value and less variance/higher precision) is assumed. According to Bayes theorem, the posterior (blue curve) is a precision-weighted combination of likelihood and prior. Thus, due to the higher and more reliable prior for breathlessness perception than breathing patterns, the resulting posterior is shifted towards higher breathlessness levels, even though sensory input (likelihood) is the same as for breathing patterns. The inferred bodily state (posterior) is thus closer to the actual sensory input in breathing patterns and closer to the prior in symptom perception. While the respiratory state is correctly inferred for breathing patterns, an incorrect internal model, e.g., with a strong weighting of erroneous priors, could lead to strong breathlessness despite an intact respiratory body state. (**B**) Alternatively, the same internal model could be used, yielding the same posterior (top, blue curve) but different cost-functions could be applied to decide on the action that should be taken. Applying a cost function mathematically corresponds to a convolution (⊗) of the posterior with the cost function. This yields the expected costs (solid line for breathing patterns, dotted line for reported breathlessness) for each possible action and allows to choose the action associated with minimum costs. An action can either be a specific breathing pattern or a symptom report. While breathing patterns and symptoms are outcomes that can be consciously perceived, the decision process involving the application of cost functions (**B**), or the formation of priors (**A**), is happening subconsciously.

We explored different computational mechanisms that could explain our observed results. Since breathing control is intact but breathlessness reports increased, one possibility would be the use of different internal models (and thus priors). For example, assuming the same sensory input and reliability, but a higher and more reliable prior for symptom perception than breathing patterns, would result in increased breathlessness reports while breathing regulation is within normal limits (see Fig. 4A). This would be in line with hierarchically arranged levels of interoceptive respiratory processing, ranging from homeostatic regulation of breathing control to meta-cognitive processes of symptom perception that are connected and share algorithmic similarities but also include different processing steps^44^.

To specifically explain the observed differences in breathlessness ratings, we further tested what kind of prior would lead to the observed results (see Appendix Figure C). We first considered the effect of a prior for higher breathlessness in patients that is equally reliable as in healthy participants. In this case, breathlessness ratings are already increased during the baseline phase with room air and progressively increase during rebreathing. Thus, the difference in breathlessness ratings persists when the stimulus strength is increased, which is in line with our results. Accordingly, one possible explanation for the observed differences in breathlessness is that patients expect higher levels of breathlessness in the experiment than controls, with both groups being similarly certain about their a-priori expectations. To explain the observed breathlessness ratings within the patient group, i.e., between patients with and without a history of breathlessness, we propose that patients with post-COVID breathlessness are more certain about their a-priori expectations of increased breathlessness during the experiment than those patients without post-COVID breathlessness. This would be represented in a similarly high but more reliable prior, which would lead to the observed increased breathlessness ratings during baseline and a decrease of this difference during rebreathing (see Appendix Figure C).

We further explored how different cost functions would influence breathlessness perception and breathing regulation (see Fig. 4B). Cost functions are an essential part of Bayesian decision theory. The brain’s inference of the underlying body state is represented as the posterior distribution. It describes the probability of the value or category of a bodily state given the current sensory input and the prior knowledge about this state. However, the brain must eventually choose one value from this posterior probability distribution. Cost functions allow to integrate rewards and costs into this decision-making process and thus decide on the action with highest expected reward (or lowest expected cost) given the current bodily state represented by the posterior distribution^45–47^. This action can either be a specific breathing behaviour or a decision to consciously perceive and report a specific symptom level. Cost functions are task specific and depend on the desired goal. A stronger perceptual response in relation to the underlying body state will lead to an earlier initiation of preventive measures that reduce further bodily stressors. Consequently, it is ‘safer’ to perceive symptoms that overrepresent the underlying body state. This would be achieved by applying an asymmetric cost function that rewards symptom reports overrepresenting the actual underlying body state and penalizes underrepresenting them. This would be similar to a better-safe-than-sorry strategy that has previously been suggested by Van den Bergh et al. (2021). This explanation assumes the same internal model for respiratory control and symptom perception, but the use of a symmetric cost function for respiratory control and an asymmetric cost function for symptom perception, leading to the observed increased breathlessness ratings despite normal breathing control. Similarly, it can explain the observed differences in breathlessness ratings between patients and healthy participants.

### Hyperventilation

While breathing patterns were similar to healthy controls in most patients, 20% of post-COVID patients hyperventilated during the experiment. Recently, carotid body dysfunction has been proposed as a possible cause of hyperventilation in post-COVID syndrome. The carotid body monitors and provides feedback about CO_2_ levels and changes in blood pH. Dysfunction could, for example, result in an over-reactive breathing response to otherwise normal pH levels^4^. A similar response could be due to dysfunctions in inspiratory muscles that has recently been shown in patients with post-COVID^48^. Pathological dysfunction on the side of the sensor (carotid body) or effector (inspiratory muscles) are a possible explanation for the observed breathing patterns and breathlessness reports in patients that hyperventilated in our experiment and could lead to erroneous processing of interoceptive respiratory signals. Six out of the eight patients that hyperventilated breathed normally during the baseline phase, and only started to hyperventilate during the recovery phase after the aversive rebreathing stimulus. Because fear of bodily symptoms in response to breathing distress has been shown to alter breathing, also this explanation may account for the observed hyperventilation responses. Stress-related hyperventilation is a type of feedforward regulation^49^ and thus overweighing priors during breathing regulation could play a role in this subgroup. A catastrophic interpretation of interoceptive signals has also been suggested to underlie the observed discordance between physiological measures and symptom reports during a hyperventilation provocation test^50^. Importantly, the dissociation between breathlessness and breathing patterns in our study remains when removing participants who hyperventilated during the experiment from the analysis.

### Internal models

Our results thus suggest that the cause of heightened symptoms in patients is not directly related to breathing physiology but rather to differences in processing and incorporating interoceptive breathing signals into symptom perception due to incorrect internal models or overly cautious cost-functions. It is currently unclear where and how internal models for interoception are represented and maintained in the brain, but there is evidence that brain areas like the anterior insula^51^ play an important role. There have been several findings of brain changes concerning the structural as well as functional connectivity in patients with post-COVID^52,53^, some of them pertaining to brain areas that are thought to be involved in interoceptive processing. Pathological changes in these brain areas could lead to problems in correctly maintaining and adapting internal models. Similarly, internal models and cost functions can be adapted by experience. For example, experiencing a persistent immune reaction due to viral reservoirs or social or emotional stress and anxiety, can lead to an adaptation of internal models that assume a high risk for aversive bodily stressors and thus the development of incorrect priors. Anxiety and depression are known risk factors for developing post-COVID symptoms^54,55^ and there is a close link between anxiety and respiratory-related interoception that is especially strong at higher levels involving meta-cognitive processes of interoception with different activities in the anterior insula in individuals with low versus high anxiety^51^.

Our computational approach provided insights into possible ways how internal model-based prior expectations and cost-functions might be altered. In specific, we have shown that our data can be explained by patients expecting a higher level of breathlessness, however, being equally certain about their prior expectation as healthy participants. Better understanding how exactly priors, i.e., (implicit) symptom expectations, are altered are crucial to develop new and improve existing therapeutical approaches. This is supported by previous studies showing that breathlessness reductions during pulmonary rehabilitation in COPD patients are associated with altered neural processes reflecting learned breathlessness associations^56^. Furthermore, virtual-reality approaches that alter breathlessness perception by modulating expectations have been proposed to re-align breathlessness with sensory signals^57^ which has implications for exposure based cognitive-behavioural therapies.

## Conclusion

In summary, we have provided first evidence that while breathing control is mostly intact, processes in the brain for symptom perception are erroneous in patients with post-COVID syndrome. We have suggested different computational mechanisms that could underlie this erroneous processing in a Bayesian brain framework. This theoretical approach allowed to investigate how prior symptom expectations must be altered to match our experimental data which can guide development of specifically targeted treatments options. Importantly, these priors are mostly implicit and a result of specific brain activity that might be altered due to disease-specific or transdiagnostic processes that are currently not well understood. Our results highlight that in addition to physiological processes in the body, further research could greatly benefit from focusing on interoception and its influence on symptom-related processing in the brain by taking a Bayesian brain perspective.

## Electronic supplementary material

Below is the link to the electronic supplementary material.

Supplementary Material 1

## Data Availability

Anonymized physiological data and breathlessness ratings recorded during the rebreathing experiment as well as lung function parameters, MOCA scores and questionnaire data (PHQ-15, CFQ, prevalence of common post-COVID symptoms, breathlessness ratings in everyday life situations) can be found via the following link: https://osf.io/vt2j5/?view_only=9c65fd05163249b2b116c750bde43476.
