# Supplementary material for: Increased breathlessness in post-COVID syndrome despite normal breathing patterns in a rebreathing challenge
Source: Sci Rep. 2025 Jul 29;15:27666. doi: 10.1038/s41598-025-11728-x (PMC12307924; doi:10.1038/s41598-025-11728-x)
Supplement: Supplementary file 1 — Supplementary Material 1 [file 41598_2025_11728_MOESM1_ESM.docx]

# Demographic characteristics

Figure A Demographic characteristics of the patient and healthy participant group for age (BF_10_ = 0.442), height (BF_10_ = 0.289) and body mass index (BMI; BF_10_ = 0.265).

# Clinical characteristics

## Chalder Fatigue Scale and Patient Healthy Questionnaire

Figure B Clinical characteristics. A) Results for Chalder Fatigue Scale (CFQ) for each question (top) and overall group comparison (bottom, BF_10_ = 3.8*10^16^). B) Results for Patient Health Questionnaire 15 (PHQ-15) for each question (top) and overall group comparison (bottom, BF_10_ = 1.79*10^12^). C) Presence and severity of current symptoms frequently reported in post-COVID syndrome.

# Instruction for rebreathing experiment

German (original)

Im folgenden Experiment atmen sie Luft mit unterschiedlichen CO_2_ Konzentrationen ein. Das kann zu Atemnot führen, muss aber nicht. Wir messen kontinuierlich ihre physiologischen Atemparamter und bitten Sie alle 10 Sekunden anzugeben, ob Sie Atemnot haben und falls ja, wie stark diese ist.

Atemnot kann bei Personen ganz unterschiedliche Empfindungen auslösen. Das kann zum Beispiel das Gefühl sein, nicht genügend Luft zu haben bzw., dass die geatmete Luft nicht ausreicht. Es kann sich in dem Wunsch oder Drang äußern, z.B. an ein Fenster zu gehen und dort frische Luft zu atmen. Es kann auch mit dem Drang verbunden sein, das Mundstück loszulassen und frei oder mehr zu atmen. Ebenso kann sein, dass Sie das Gefühl haben, dass die aktuelle Atmung nicht ausreicht.

Atemnot kann auch mit einer gewissen Anstrengung oder Schwierigkeiten bei der Atmung verbunden sein. Diese Empfindungen können auftreten, müssen aber nicht und die Empfindungen können kommen und gehen. Atemnot kann also ganz unterschiedliche Empfindungen auslösen und diese können in unserem Experiment kommen und gehen oder gar nicht auftreten.

English (translation)

In the following experiment, you will breathe air with different CO_2_ concentrations. This can lead to breathlessness but does not have to. We continuously measure your physiological breathing parameters and ask you to indicate every 10 seconds whether you experience breathlessness and, if so, how strong it is.

Breathlessness can trigger very different sensations in people. For example, it can be the feeling of not having enough air or that the air you are breathing is not enough. It can manifest itself in the desire or urge to go to a window and breathe fresh air, for example. It can also be associated with the urge to let go of the mouthpiece and breathe freely or more deeply. You may also feel that your current breathing is not sufficient.

Breathlessness may also be associated with a certain effort or difficulty in breathing. These sensations may or may not occur and the sensations may come and go. Breathlessness can therefore trigger very different sensations and these can come and go or not occur at all in our experiment.

# Influence of different priors on breathlessness perception

Figure C The effect of different priors (i.e., different internal models) on breathlessness reports. Top: During rebreathing sensory input and thus the mean of the likelihood function increases. Since lung function is intact, the same likelihood function is assumed for patients and healthy participants. Second row: The internal model for healthy participants leads to a prior with a low mean to which low weight is assigned (high variance, i.e., low precision of the prior). Thus, healthy participants expect low breathlessness, but rely mostly on sensory input. Third row: Patients without breathlessness assume a higher breathlessness level than healthy participants but are equally certain about their belief. The prior variance is thus the same as in healthy control participants, but the prior is shifted to the right, i.e. higher breathlessness levels. This leads to a shift of the posterior towards higher breathlessness levels than in healthy participants. When the stimulus strength increases (likelihood 2 in the top panel), the shift of the mean of the posterior is equally strong as in healthy participants, i.e., breathlessness differences during rebreathing remain (same length of errors in panel 2 and 3). Bottom: Patients with breathlessness expect an equally high level of breathlessness but are more certain about their belief than patients without breathlessness. This leads to a lower variance, i.e., higher precision of the prior. During phases with low stimulus intensity this will lead to a higher posterior mean, i.e., slightly increased breathlessness reports, however, when stimulus intensity increases (e.g., during rebreathing) breathlessness reports are similar. This is in line with our experimental results.

# Comparison between frequentist and Bayesian ANOVA

Table A Comparison between frequentist (p-value) and Bayesian (BF_10_) repeated-measures ANOVA for the main analysis (including N = 40 patients and N = 40 healthy participants). .

|  | Baseline  BF_10_ | p-value | | Rebreathing  BF_10_ | p-value | Recovery  BF_10_ | p-value |
| --- | --- | --- | --- | --- | --- | --- | --- |
| Breathlessness | 8.029 | | 0.004 | 11636 | < 0.001 | 43662 | < 0.001 |
| Respiration rate | 0.817 | | 0.197 | 2.007 | 0.027 | 1.877 | 0.036 |
| Tidal volume | 0.617 | | 0.649 | 0.361 | 0.867 | 0.423 | 0.743 |
| FetCO2 | 0.695 | | 0.484 | 0.437 | 0.348 | 5.018 | 0.008 |
| Heart rate | 0.671 | | 0.504 | 0.708 | 0.287 | 0.776 | 0.135 |
|  |  | |  |  |  |  |  |

# Deviations from pre-registered analysis plan

As in our pre-registration, our experimental paradigm consisted of the following conditions:

| Breathing condition | Duration |
| --- | --- |
| Baseline (room air) | 60s |
| Rebreathing | 150s |
| Recovery (room air) | 150s |
| Cognitive Cue (room air) | 30s |
| Second rebreathing | 30s |

Since the second rebreathing phase was too short for breathlessness changes to occur and for better comparability with previous studies, we only evaluated data up to the end of the recovery phase in this paper. In our pre-registration we also included a co-variate in the repeated-measures ANOVA. Starting from the rebreathing phase, we planned to add the mean of respective dependent variable (CO_2_ concentration, breathlessness rating or breathing flow) over the last 30s of the previous breathing condition as a covariate. However, for this paper we were interested in the difference for each specific breathing condition, independent of baseline levels. We thus decided to not include the co-variate in the analysis.
